# Positioning a proned patient with cauda equina syndrome who presents at 15 weeks gestation: a case report

**DOI:** 10.12688/f1000research.3310.1

**Published:** 2014-05-27

**Authors:** Elizabeth Speirs, Matthew Wiles, Andrew Bacon, Stephen Radley

**Affiliations:** 1 Royal Hallamshire Hospital, Sheffield Teaching Hospitals, Sheffield, S10 2JF, UK

## Abstract

Cauda equina syndrome is a neurosurgical emergency that requires prompt intervention to prevent irreversible spinal cord paralysis. This article describes how we managed a case of an obese pregnant patient who was placed in the prone position for surgery. We discuss the evidence behind the management options and choice of operating tables available.

## Case

We present a case of Cauda equina syndrome in a 24 year old woman at 15 weeks gestation. She had no other medical problems to note and had previously had two uncomplicated pregnancies. Her elevated body mass index (BMI) of 36 kg/m ^-2^ did provide a potential difficulty in managing this case.

**Figure 1.**
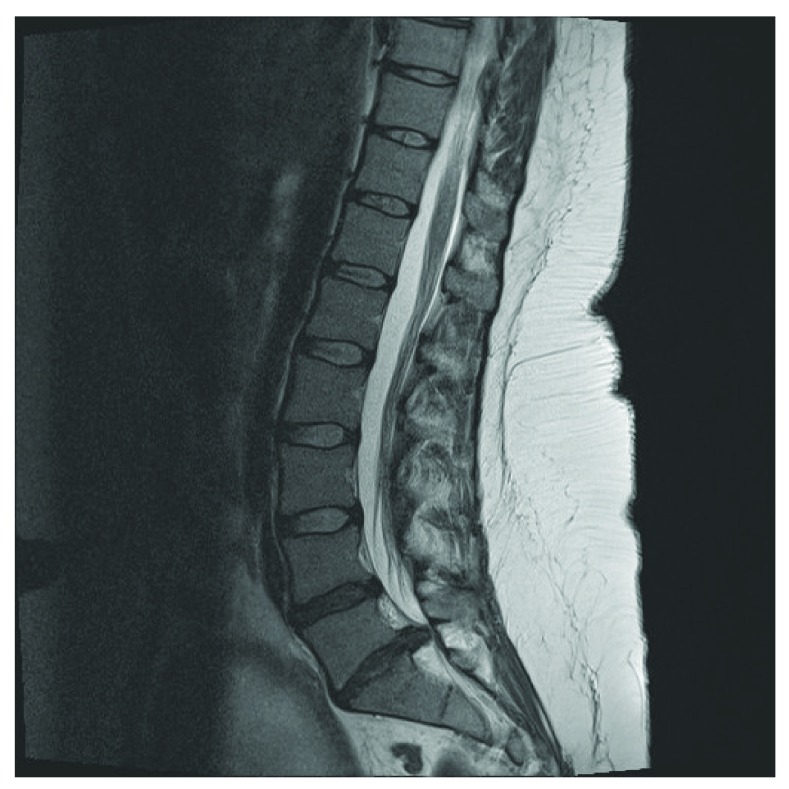
Pre-operative MRI lumbar spine showing a L5/S1 prolapse.

The patient was referred to our tertiary neurosurgical unit with a 24 hour history of severe lower back pain, altered perianal sensation and right lower limb weakness. An urgent MRI scan showed a large L5/S1 disc prolapse ( Figure 1). A frank discussion of the risks of medical versus surgical management was undertaken, and immediate surgery was chosen by the patient.

The decompression was performed with our patient proned under general anaesthesia, which was maintained using a combination of sevoflurane and remifentanil. Due to the body habitus of the patient (BMI 36 kg/m ^-2^), it was felt that undertaking surgery in the lateral position would have been technically difficult, with an increased operative time and a greater risk of bleeding. We chose to prone the patient on to a Jackson table (OSI, Union City, CA) with supports on the sternum and laterally on the iliac spines. Absolute care was taken to ensure that there was no abdominal compression once positioned.

The operation was performed uneventfully and lasted 90 minutes. Cardiovascular stability was maintained throughout. The patient’s neurology had fully recovered by the first postoperative day and a fetal ultrasound that day showed a viable fetus. Unfortunately this was later found to be a twin pregnancy, one of whom was spontaneously aborted. The second twin was delivered healthy at 39 weeks gestation.

## Discussion

There is little literature currently available to guide positioning, especially discussing proning techniques in pregnant patients for spinal surgery. Discectomies in gestational patients can be performed in both the prone and lateral position ^1^. A proned patient allows better surgical access, but the lateral position may make it easier to ensure that there is no abdominal compression to compromise uterine blood flow. Even though the majority of operating tables designed for proning allow the abdomen to hang free in non-pregnant patients, they are not designed to fit the larger pregnant abdomen. We could find no literature comparing the use of different operating tables and the abdominal space that they offer a pregnant abdomen.

Uterine blood flow during prone positioning has been examined by Nakai *et al.* ^2^. They took 23 healthy women at 34 weeks gestation and laid them in a supine, right lateral, left lateral and prone position. The proning table had a hole in it for the pregnant abdomen so they could see that no compression occurred. They found that prone positioning actually provided optimal relief of umbilical artery compression as measured by the umbilical artery systolic/diastolic ratio, and that this is superior to that of both the right and left lateral positions. The effect of the prone position on uterine blood flow in an anaesthetised patient has not been determined however.

The few small case series and cases that we found describing spinal surgery in the prone position for gestational women have demonstrated good fetal outcomes. One series ^3^ presents the course of three women of 16 to 20 weeks gestation who required discectomies for lumbar disc herniation during pregnancy. They all received epidural anaesthesia and then self positioned themselves prone on a Relton-Hall laminectomy frame (IS, Dorval, Quebec). All babies were delivered without complications at full term. The authors chose the Relton-Hall frame as it has pressure points on the anterior superior iliac spines and the chest, which they felt allowed most freedom of the abdomen and uncompressed Inferior vena cava blood flow. In addition, they concluded that letting the women position themselves prior to receiving an anaesthetic was an extra safety measure. They believed that if they could ensure that the women were comfortable once proned, they were satisfied that there was no undue pressure on the abdomen and uterus. Even at 30 weeks gestation, the technique of regional anaesthesia and patient self-positioning has been seen to have no adverse foetal effects ^4^. In this case they also undertook foetal monitoring throughout surgery, and noted no foetal compromise intra-operatively.

Cervical and thoracic decompressions for epidural haematomas are similar surgical emergencies that present the need to prone patients under general anaesthesia. A case series of six patients reports all the babies being born healthily after spinal decompressions ^5^. These patients were placed on a Wilson frame (ZA, Jiangsu, China). Three procedures were undertaken at 20, 24 and 34 weeks, with spontaneous deliveries occurring at term. Three other women of 35, 38 and 41 weeks gestation underwent caesarean sections prior to decompression with no adverse foetal outcomes. The recommendations from this series are that neurosurgical intervention to avoid permanent neurological damage is safe and that surgery should not be delayed in obstetric patients.

There is only one published guideline that we could find on positioning and spinal surgery in gestational women, produced by a centre in Korea ^1^. They followed a case series of ten women from their institution, 6 of who had lumbar disc herniation, the others infection or tumour. All patients in the first trimester were placed in a prone position, five delivering healthy full term babies whilst one patient had a therapeutic abortion for early radiation exposure. Of the later term patients, two were put in right, and two in left lateral positions. One baby was born healthily at full term, one by elective caesarean section at 34 weeks gestation, one pre-term at 33 weeks spontaneously and one therapeutic abortion was performed, also due to radiation exposure. Their guidelines produced from this review suggest epidural anaesthesia for most procedures, but for longer operating duration general anaesthesia may be preferable. It also recommends that during the first and early second trimester a prone position for surgery is safe, but a left lateral position is preferable for the latter part of the second trimester and third trimester. They conclude that from their review and own experiences peripartum, neurosurgical procedures can be safely performed in most pregnancies.

## Conclusion

In summary, there is very limited evidence to guide the optimal management of the pregnant patient who requires emergency spinal surgery. However, safe outcomes for both mother and fetus undergoing general anaesthesia for varying pathologies during the first trimester have been seen in many case series ^6^. These women were all supine, but there appears to be no reason to delay general anaesthesia during the first trimester. For pregnancies of 34 weeks gestation and over, a caesarean section can be performed safely prior to, or, as a combined procedure with discectomies ^7^.

Another very important aspect of case management involves a multi disciplinary team approach peri-operatively. A fetal ultra sound should be performed before any intervention is undertaken and fetal heart monitoring intra-operatively should be considered. Alterations in fetal heart rate can alert the anaesthetist as early as possible to aortocaval compression and cardiovascular insufficiency due to poor positioning of the mother. Close obstetric follow up is essential in any non-obstetric surgery.

From all the literature that we have looked at, there appears to be no reason to avoid the prone position for surgery. Nor is there evidence to suggest that regional or general anaesthesia offers significant benefits to either mother or foetus. There is also no particular operating table that we can recommend, but knowledge of your own equipment is vital. We believe that the key aspects of the management of such cases is to take meticulous care during positioning to ensure that the abdomen is free, no matter what position or operating table is chosen.

## Consent

Written informed consent for publication of their clinical details and/or clinical images was obtained from the patient.
