# Supplementary material for: Positioning a proned patient with cauda equina syndrome who presents at 15 weeks gestation: a case report
Source: F1000Res. 2014 May 27;3:117. [Version 1] doi: 10.12688/f1000research.3310.1 (PMC4111115; doi:10.12688/f1000research.3310.1)
Supplement: Peer review history

# Peer review: Referee response for version 1

**DOI:** 10.5256/f1000research.3551.r4903

**Published:** 2014-06-11

**Authors:** Michael Paech, Lars Wang

**Affiliations:** 1 Department of Anaesthesia and Pain Medicine, The University of Western Australia, Perth, WA, Australia; 2 Royal Perth Hospital, Perth, WA, Australia

The submission is a case report of a 15 week pregnant woman who needs urgent lumbar discectomy for cord compression. This requires the use of the prone position (PP) on a Jackson table.

The use of a so-called Jackson Table is sensible. It allows for the abdomen to hang freely between the pelvic and chest support. It thus reduces the epidural venous pressure that may cause troublesome bleeding for the surgeon. The Wilson frame (as mentioned in the Discussion) will cause more pressure on the abdomen.

The main message is the discussion of the effects of the PP on uteroplacental blood flow and fetal outcome. However, in week 15 the effects on the uteroplacental blood flow may not be significant and the other problems associated with the prone position should be mentioned.

A number of other case reports and case series are presented that indicate the PP may be safe during pregnancy. However, the lack of reference to the anaesthetic literature in the references suggests that the authors may not be aware use of the PP during GA is already reasonably well described during pregnancy.

More specific comments:

- The word ‘proning’ (and term ‘proned patient’ ) are incorrect in our opinion – use as a verb except in gaming vernacular. The terms ‘prone’ or ‘patient in the prone position’ should be substituted throughout.
- There is inadequate discussion of the anaesthetic issues. Although “safe outcomes” occur after GA in the first trimester, the clear consensus in the anaesthesia community is to avoid or delay GA until the second trimester if possible and to use regional anaesthesia when possible.
- **Conclusion:**
**L2-3: **What is meant by “varying pathologies”? Do the authors refer to spinal pathology or any pathology that requires general anaesthesia during first trimester?
**L7-8:** Fetal heart rate monitoring was not adequately discussed. During surgery is not useful until after fetal viability and is difficult to interpret at early gestations. It may give some indication of fetal compromise that prompts change in anaesthesia, but it is only of true value if provision has been made to perform emergency delivery, which does not apply in this case.

We have read this submission. We believe that we have an appropriate level of expertise to confirm that it is of an acceptable scientific standard, however we have significant reservations, as outlined above.

# Peer review: Referee response for version 1

**DOI:** 10.5256/f1000research.3551.r4956

**Published:** 2014-06-02

**Authors:** John Svigos

**Affiliations:** 1 Discipline of Obstetrics & Gynaecology, School of Paediatrics & Reproductive Health, Faculty of Health Sciences, University of Adelaide, Adelaide, SA, Australia

Whilst I find it to be an informative and current paper with a good appraisal of the available literature, intuitively and grammatically I believe the use of "proning" etc is incorrect as prone is an adjective and must be used in this manner as the "alternative" use detracts from the paper.

I have read this submission. I believe that I have an appropriate level of expertise to confirm that it is of an acceptable scientific standard.

# Peer review: Referee response for version 1

**DOI:** 10.5256/f1000research.3551.r4905

**Published:** 2014-06-02

**Authors:** Bernard Wittels

**Affiliations:** 1 Continental Anesthesia, Saint Francis Hospital, Evanston, IL, USA

These authors present an unusual case of an obese, pregnant woman at 13 weeks gestational age who presented with perineal hypesthesia and partial loss of motor function in her right lower leg. An MRI showed an L5-S1 herniated nucleus pulposus that required emergency decompression. The authors discuss their anesthetic management of the patient and review the current literature.

**Title:** The title should be re-worded because the patient did not have cauda equina syndrome (which usually includes partial or full loss of control of bowel and bladder sphincters, and may include painful paresthesias), and the word “prone” should only be used as an adjective. A more appropriate title would be: *Anesthesia for emergent lumbosacral discectomy and nerve decompression in an obese parturient at 13 weeks gestation*.

**Case report**:

1. Again, the term cauda equina should be removed. The list of potential difficulties in administering anesthesia to an obese, preterm parturient needs to be elaborated in detail. There is no mention of consenting the patient for the MRI, including the risks of MRI to the developing fetus. The patient's sensorimotor radiculopathies represent a neurosurgical emergency; medical therapy in this setting does not conform to the standard of care and should not have been offered as a valid alternative therapy. To conform with their discussion points, the authors should have obtained an obstetric consultative evaluation. The absence of preoperative, intraoperative, and postoperative obstetric consultations should be explained. The failure to detect twin gestations at 13 weeks of age, despite having an MRI, needs to be discussed.
2. It appears that epidural and/or spinal anesthesia were not considered or offered as an alternative to general anesthesia, nor were these two anesthesia techniques compared for their relative risks and benefits to the mother and her fetus. The authors should mention that spinal anesthesia produces decreased fetal drug exposure and decreased maternal blood loss, compared to general anesthesia. What was the patient's airway evaluation? Many obese parturients have difficult airways that can pose life-threatening problems with hypoxemia and failure to intubate after induction of anesthesia. How was the anesthetic induction managed? What drugs were used? What happened to her oxygenation, ventilation, and hemodynamics on induction of anesthesia? Why were sevoflurane and remifentanil chosen for maintenance of anesthesia? What advantages or disadvantages does this regimen have over other choices?
3.  In an obese patient, the prone position would seem to be the only reasonable choice by the neurosurgeon. Abdominal compression is just one consideration in the safe positioning of an obese, pregnant patient prone on the operating table. What are the other concerns? Could the fetal heart rate be monitored externally? If so, then why wasn't fetal heart monitoring employed intraoperatively? What would be the expected fetal heart rate pattern?
4. Exactly when was the twin gestation diagnosis made? Why wasn't that diagnosis made earlier? Could the radiation from the MRI, or the effects of surgery, anesthetic drugs, or the prone position have any potential effect on halting the growth and development of the unborn twin?

 

**Discussion:** It would be useful for the authors to distinguish known safety concerns in this setting from factors that are unknown and untested. This section should be shortened. The Nakai reference should be dropped because it was done in awake patients near term gestation. No comparative study of OR tables for the prone position of pregnant patients has been done. It is inconsistent to review a study that used intraoperative fetal monitoring without commenting on why it was not used on your study case patient. None of the referenced studies involved preterm gestations or obese parturients. ASA guidelines are worth referencing, but not one from Korea based on a single study of ten patients.

**Conclusion:** What did you do differently throughout the perioperative period to manage these problems that were unique to your case? Where did you succeed? After reviewing the literature, how do you see your case enhancing our knowledge of neuroanesthesia during pregnancy? Since there are no studies comparing regional versus general anesthesia in this setting, no conclusion can be made about their relative benefits and risks. Abdominal compression alone is not the only major concern with the prone position. What are other concerns and how are these assessed and managed? What would you do differently or better in the future to provide the safest level of care? Can you suggest further research that is needed?

I have read this submission. I believe that I have an appropriate level of expertise to confirm that it is of an acceptable scientific standard, however I have significant reservations, as outlined above.
